# Diabetic nephropathy associates with deregulation of enzymes involved in kidney sulphur metabolism

**DOI:** 10.1111/jcmm.15855

**Published:** 2020-09-16

**Authors:** Elena Uyy, Viorel Iulian Suica, Raluca Maria Boteanu, Florentina Safciuc, Aurel Cerveanu‐Hogas, Luminita Ivan, Crina Stavaru, Maya Simionescu, Felicia Antohe

**Affiliations:** ^1^ Institute of Cellular Biology and Pathology “Nicolae Simionescu” Bucharest Romania; ^2^ “Cantacuzino” National Institute of Research and Development for Microbiology and Immunology Bucharest Romania

**Keywords:** diabetes, early nephropathy, proteome, sulphur metabolism

## Abstract

Nephropathy is a major chronic complication of diabetes. A crucial role in renal pathophysiology is played by hydrogen sulphide (H_2_S) that is produced excessively by the kidney; however, the data regarding H_2_S bioavailability are inconsistent. We hypothesize that early type 1 diabetes (T1D) increases H_2_S production by a mechanism involving hyperglycaemia‐induced alterations in sulphur metabolism. Plasma and kidney tissue collected from T1D double transgenic mice were subjected to mass spectrometry‐based proteomic analysis, and the results were validated by immunological and gene expression assays.T1D mice exhibited a high concentration of H_2_S in the plasma and kidney tissue and histological, showed signs of subtle kidney fibrosis, characteristic for early renal disease. The shotgun proteomic analyses disclosed that the level of enzymes implicated in *sulphate activation modulators, H_2_S‐oxidation* and *H_2_S‐production* were significantly affected (ie 6 up‐regulated and 4 down‐regulated). Gene expression results corroborated well with the proteomic data. Dysregulation of H_2_S enzymes underly the changes occurring in H_2_S production, which in turn could play a key role in the initiation of renal disease. The new findings lead to a novel target in the therapy of diabetic nephropathy. Mass spectrometry data are available via ProteomeXchange with identifier PXD018053.

## 1 INTRODUCTION

Type 1 diabetes (T1D) are characterized by a targeted autoimmune destruction of the insulin‐producing β‐cells in the pancreatic islets of Langerhans and the ensuing insufficient insulin production. The different degrees of β‐cell dysfunction lead to chronic hyperglycaemia accompanied by an inflammatory reaction and immune responses that affect major organs including eyes, nerves, heart, blood vessels and kidneys. A major feature of diabetic kidney disease is the apoptosis of tubular epithelial cells as a result of increased free radicals production and amplified oxidative stress under the hyperglycaemic condition.^1^ Most of the current studies on T1D use animal models that promptly develop severe disease after administration of diabetogenic substances, such as streptozotocin or alloxan.^2^ To address the early changes occurring in diabetic nephropathy, we chose to employ double transgenic diabetic mice (dTg) that, between the 3rd and the 8th week of age, develops gradually T1D with somatic, metabolic and vascular disorders comparable to human diabetes.^3^, ^4^, ^5^ These characteristics allow the study of the etiopatogenic mechanisms of early diabetic nephropathy, the understanding of which could open up the possibility of finding new targets for the therapy of this disease.

Hydrogen sulphide (H_2_S) is produced redundantly by the kidney and has a crucial role in renal physiology (ie regulation of the excretory function of the kidney, release of renin and oxygen sensor) and pathology (ie renal ischaemia/reperfusion, obstructive or hypertensive nephropathy). H_2_S is a colourless, water‐soluble and membrane‐permeable gas, considered the third gaseous signalling molecule after nitric oxide and carbon monoxide. It is generated through the cysteine metabolism by cystathionine b‐synthase (Cbs) and cystathionine g‐lyase (Cth) enzymes or by the concerted action of cysteine aminotransferase, D‐amino acid oxidase (Dao) and 3‐mercaptopyruvate sulphurtransferase (Mpst). Recent studies reveal a complex role for H_2_S in many biochemical processes taking place during angiogenesis and carcinogenesis. H_2_S has a protective effect being a potent antioxidant, anti‐apoptotic, anti‐inflammatory and oxidative stress regulator.^6^

In streptozotocin‐treated murine animal models, H_2_S has been reported as a protective molecule, playing a key role in the reduction of endothelial dysfunction, cardiomyopathy and nephropathy.^7^, ^8^, ^9^, ^10^, ^11^ Addressing the role of H_2_S in diabetes, Xie et al^7^ demonstrated for the first time that H_2_S suppresses diabetes‐induced accelerated atherosclerosis.^12^, ^13^

However, there are no consistent data on the H_2_S role and its concentration in the plasma and different tissues of the diabetic patients and experimental animal models.^14^, ^15^ Some studies argue that the plasma H_2_S level in streptozotocin‐induced diabetic rats is reduced^16^ while others, on similar animal models report unchanged plasma levels of H_2_S, although insulin administration results in increased plasma H_2_S concentration.^15^

Thus, due to its importance, we examined the changes occurring in the plasma and kidney concentration of H_2_S level in early T1D and questioned the underlying mechanisms of this alteration. We hypothesize that hyperglycaemia deregulates the enzymes involved in H_2_S metabolism, which in turn change the equilibrium of H_2_S production that could play an important role in the onset of early kidney dysfunction in diabetes.

To test this hypothesis, we designed experiments using a double transgenic diabetic mouse model and high‐performance mass spectrometry‐based proteomic analysis. Hereby, we present data showing that in diabetes there's an increase in circulating H_2_S and the diabetic kidney exhibits significantly differentially expressed enzymes implicated in sulphur metabolism. The results provide new markers of kidney dysfunction that could become novel therapeutic targets in T1D complications.

## 2 MATERIALS AND METHODS

All chemicals used for liquid chromatography (LC) and mass spectrometry (MS) experiments were of LC‐MS grade. Trypsin Gold was purchased from Promega. Urea, sodium deoxycholate (DOC), Trizma hydrochloride (Tris), DL‐dithiothreitol (DTT), iodoacetamide (IAA), N‐acetyl‐L‐cysteine (NAC), ethylenediaminetetraacetic acid (EDTA), bovine serum albumin (BSA), ammonium bicarbonate and all solvents were provided by Sigma‐Aldrich, unless otherwise specified. Complete protease inhibitor cocktail was offered by Roche. C18 solid‐phase extraction (SPE) columns were acquired from Waters Corporation. Protein concentration was determined by amido black assay using Amido Black 10B (JT Baker Chemical Co.). Cholesterol, glucose and triglyceride plasma levels were determined using the specific assays from DIALAB GMBH. The concentration of renal plasma and renal tissue H_2_S were determined by the H_2_S Assay Kit E‐BC‐K355 (Elabscience) according to the manufacturer's instructions.

### 2.1 Experimental animal models

Twenty transgenic mice were from the ‘Cantacuzino’ National Institute of Research and Development for Microbiology and Immunology (Bucharest, Romania) and housed in suitable facility under controlled temperature, humidity and 12 hours light cycle. The Ins‐HA^±^/TCR‐HA^±^ double transgenic Balb/c mice (dTg) were obtained as previously described.^3^, ^4^ Briefly, the mice express simultaneously the hemagglutinin (HA) of influenza virus under the rat insulin promoter (Ins‐HA) and T cell receptor (TCR) specific for the immunodominant CD4 T cell epitope of HA (HA110‐120) in pancreatic β‐cells. Since the Ins‐HA^+/+^Tg mice were homozygous and the TCR‐HA^±^ Tg mice were heterozygous, the offspring were either Ins–HA^±^/TCR‐HA^±^ (dTg) mice (which developed diabetes) or Ins–HA^±^/TCR‐HA^‐/‐^ (sTg) mice (which did not develop diabetes). As controls, we employed both, the single transgenic, Ins–HA^±^/TCR‐HA^‐/‐^ (sTg) and the wild‐type Balb/c mice (WT). The animals were divided in three experimental groups, ten animals per group: (a) double transgenic mice (dTg) that developed autoimmune T1D; (b) single transgenic mice (sTg); and (c) BALB/c mice (WT). During the experiments, the animals had free access to standard diet and freshwater. After 12 weeks, the animals were evaluated for changes in the bodyweight and plasma glucose concentration. When hyperglycaemia reached high level in the dTg group (435 ± 108 mg/dL), the mice were anesthetized via an i.p. injection with a ketamine (100 mg/kg bodyweight)/xylazine (10 mg/kg bodyweight) cocktail for mice. After the blood was collected on 5 mmol/L EDTA through a ventricular puncture, the mice were perfused with phosphate buffered saline (PBS) and the kidneys were harvested and processed for further experimental assays. All animal experiments were conducted in accordance with ‘International Guiding Principles for Biomedical Research Involving Animals’ (Council for the International Organizations of Medical Sciences, December 2012), and regulations of the ethic Committee of ICBP ‘Nicolae Simionescu’ (approval number 373/27.06.2014).

### 2.2 Histological studies

Immediately after the standard cardiac perfusion with PBS, the left kidney of each animal was fixed by immersion in 10% formalin for paraffin embedding. The 6‐µm‐thick sections, obtained with a Leica RM2245 microtome, were double stained, for cell nuclei with a modified haematoxylin solution and for cell structures with a modified 1% eosin solution (H&E Fast Staining Kit, Carl Roth GmbH + Co. KG). For detection of collagen, Masson's trichrome staining (Carl Roth GmbH + Co. KG) was used. The sections were mounted in 90% glycerol, examined on an AxioVert.A1 inverted microscope (Carl Zeiss GmbH) and analysed with Zen Pro 2012 Software (Carl Zeiss, GmbH).

### 2.3 Protein extraction

From each animal, thirty milligrams of renal cortex tissue was mechanically homogenized (1 minutes) on ice in 0.3 mL buffer containing 8 M urea, 1% DOC and 100 mmol/L Tris‐HCl (pH 7.5) and the samples were thereafter centrifuged (11 000 ×*g*) at 4°C for 10 minutes. The supernatants were collected and stored at −30°C until use. All protein extracts were subsequently used for biochemical, mass spectrometry and Western blot (WB) quantification experiments.

### 2.4 Preparation of kidney tissue samples for mass spectrometry analysis

After solubilization, 50 µg of proteins from each sample was further purified by acetone precipitation and incubated for 60 minutes at −20°C. The cysteine residues were processed in a reducing buffer (containing 8 M urea, 0.1 M Tris‐HCl, 0.1 mol/L EDTA and 20 mmol/L DTT, pH 8.8) for 60 minutes, followed by alkylation using 80 mmol/L IAA in 0.1 M Tris‐HCl and 0.1 mmol/L EDTA buffer, for 90 minutes, in the dark, with constant agitation (600rpm) at room temperature. Quenching was done using 80 mmol/L NAC in 0.1 M Tris‐HCl and 0.1 mmol/L EDTA buffer, by shaking for 30 minues, at room temperature.

Proteolysis was performed overnight, at 37°C, using a 1:20 trypsin to substrate quantity ratio. Formic acid was added to the resulted peptide mixtures up to pH 2.5 for enzyme inhibition and DOC precipitation. The sedimented ionic detergent was discarded by centrifugation for 20 minutes, 20 000 ×*g* at room temperature. The peptides were desalted using SPE and concentrated with the Concentrator plus system (Eppendorf).

### 2.5 LC‐MS/MS analysis

LC‐MS/MS experiments were performed using an EASY–nLC II nano‐liquid chromatography system coupled to an LTQVelos Pro Orbitrap mass spectrometer (Thermo Scientific). For chromatographic separation, a trap column (EASY column, 2 cm × 100 μm.d., C18, 5 μm, 120 Å, Thermo Scientific) was connected to the analytical column (EASY column, 10 cm × 75 μm.d., C18, 3 μm, 120 Å, Thermo Scientific) and a 90 minute, 3%‐25% solvent B (acetonitrile with 0.1% [v/v] formic acid) over A (water with 0.1% [v/v] formic acid) separation gradient at a flow rate of 300 nL/min was applied. The MS was operated in a top 12 data‐dependent configuration at 60k resolving power for a full scan across the 350‐1700 m/z domain and collision‐induced dissociation (CID) fragmentation mode for MS2. Each sample was injected in triplicate.

### 2.6 Database protein identification, label‐free quantification and data mining

Protein identification was performed using Proteome Discoverer 1.4 (Thermo Scientific) and Mascot 2.4.1 (Matrix Science), and the taxonomy was set on Mus musculus organism in UniProtKB/Swiss‐Prot fasta database, build 11.2017. A maximum of 2 missed cleavage sites was allowed. Oxidation of methionine and deamidation of asparagine and glutamine were enabled as variable modifications while carbamidomethylation of cysteine was set as fixed modification. False discovery rate target was set below 0.05. SIEVE 2.1 software (Thermo Scientific) was used for label‐free relative quantification of all identified proteins. A *t* test was employed to calculate p‐value and to detect significant regulations (dTg/WT ˃1.5 or sTg/WT ˃1.5 or and dTg/WT ˂0.67 or sTg/WT ˂0.67 and *P*‐value ˂.05) over ten biological replicates. Protein Center v 3.12 bioinformatics platform was utilized for projection of the quantitative data onto Kyoto Encyclopedia of Genes and Genomes (KEGG) signalling pathways and determine their over‐representation (FDR *P*‐value <0.05). Proteins identified with a high Mascot score (>100) in the over‐represented KEGG signalling pathways (FDR *P*‐value <2.04E‐02) in diabetic samples were further used for validation using as alternative methods, Western blot (WB) and gene expression assays.

### 2.7 Western blotting assay

Equal amounts of the renal tissue protein were loaded and separated by electrophoresis using 12.5% SDS polyacrylamide gels (SDS‐PAGE). The proteins were subsequently transferred onto nitrocellulose membrane and analysed by Western blotting (WB) assay. The membranes were first exposed (2 hours) to the following primary antibodies: Cystathionine beta‐synthase (Cbs mouse monoclonal, MA5‐17273—Thermo Fisher Scientific, dilution 1:1000), sulphite oxidase, mitochondrial (Suox rabbit polyclonal, PA5‐21705—Thermo Fisher Scientific, dilution 1:1000), Thiosulphate sulphurtransferase (Tst rabbit monoclonal, ab166625—Abcam, dilution 1:1000) and β‐actin (mouse monoclonal, ab6276—Abcam, dilution 1:1000) in TBS with 1% BSA. This was followed by the appropriate secondary antibodies (1 hour), IgG coupled with horseradish peroxidase (IgG–HRP). Beta‐actin was used to normalize the semi‐quantitative data. The reaction product was detected with the ECL Western blotting Substrate kit, and the protein bands were digitally detected (ImageQuant LAS4000) and quantified by densitometry with Scion Image software.

### 2.8 Gene expression analysis

Total RNA was extracted from kidney tissues using the RNeasy Mini Kit (Qiagen). Its quality was assessed using Agilent 2100 Bioanalyzer (Agilent Technologies) and quantified by NanoDrop ND 1000 spectrophotometer (Thermo Scientific). The cDNA was generated from 1 μg total RNA using Transcriptor First Strand cDNA Synthesis Kit (Roche). Light Cycler 480 SYBR Green I Master mix was used to perform real‐time PCR analysis in the Light Cycler System (Roche). All reactions were performed in triplicate, and the product specificity was validated by melting‐curve analysis. Amplification of housekeeping gene b‐actin was used for normalization. The primer sequences used were as follows: for Cbs 5′‐GCTGGAACCTGCTCCTTTTC‐3′ (forward) and 5′‐TGCATGTCCAAGTGCTGGAA‐3′ (reverse); for Soux 5′‐GTCATGGGGACCCTGTTAGG‐3′ (forward) and.

5′‐ACATCCGTGGTGACTCCTGA‐3′ (reverse); for alpha‐actin.

5′‐AGTGTGACGTTGACATCCGTA‐3′ (forward) and.

5′‐GCCAGAGCAGTAATCTCCTTCT‐3′ (reverse). The relative quantification of gene expression was done with LightCycler^®^ 480 Software using the Ε (Efficiency)‐Method.

### 2.9 Statistics

All the data were generated by running samples in triplicate and expressed as a mean ± SD. The results were analysed by Student's unpaired *t* test using GraphPad Prism 5.0 statistical software (GraphPad Software). Significance was defined as a *P*‐value <.05.

## 3 RESULTS

### 3.1 Validation of the diabetic animal mode

**FIGURE 1:**
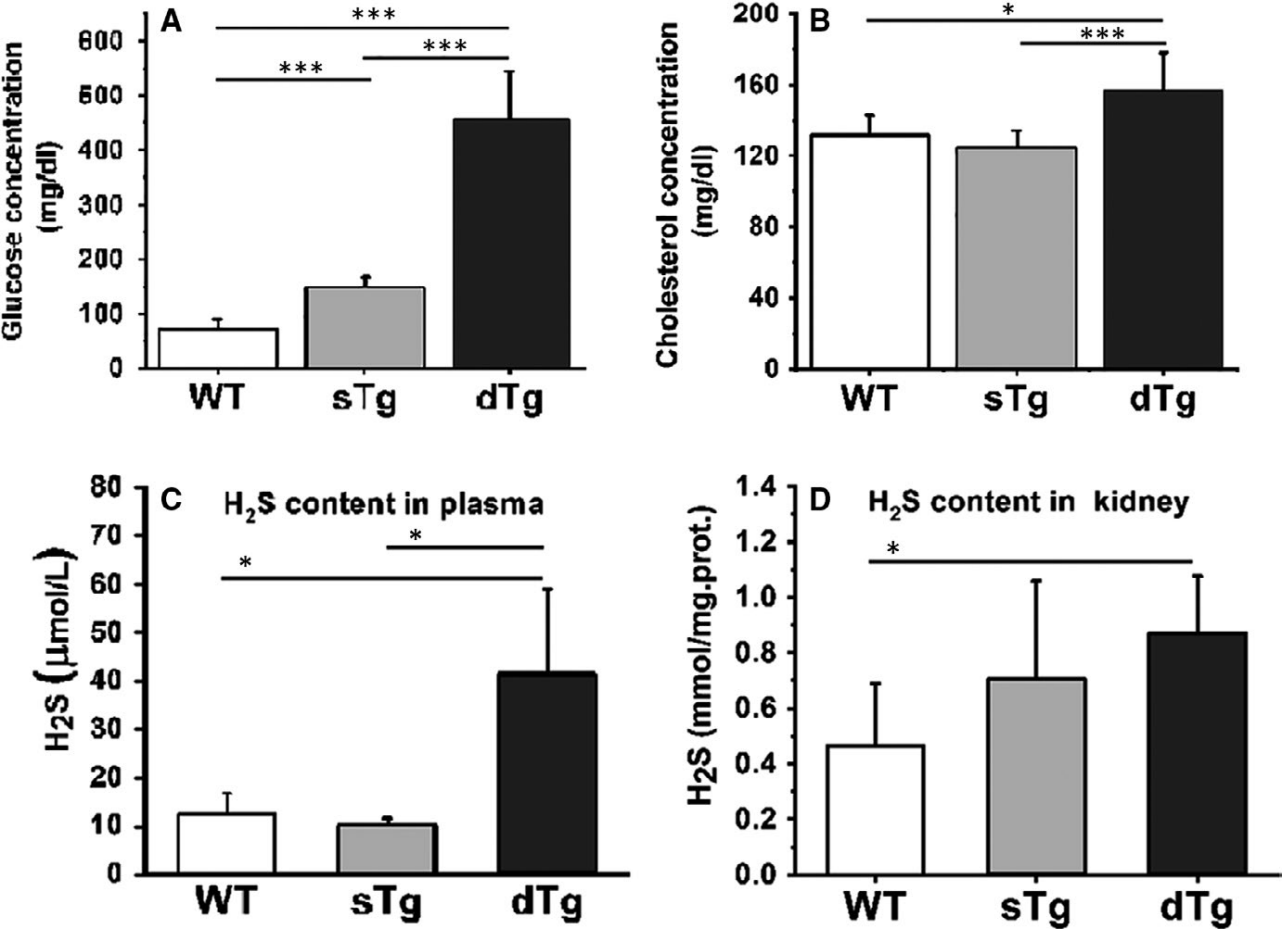
Validation of the diabetic double transgenic mice (dTg) model compared to controls, the non‐diabetic single transgenic (sTg) and BALB/C wild‐type (WT) mice. Plasma glucose (A) and cholesterol measurements (B) performed for each experimental group. H_2_S levels in the plasma (C) and the kidney tissue homogenate (D) from WT, sTg and dTg mice, respectively. Note that, type 1 diabetes is accompanied by changes of H_2_S concentration in the plasma and the renal tissue. Data are expressed as means ± standard deviation (SD); *P ˂ .05, **P ˂ .01 and ***P ˂ .001

At 13.5 weeks of age, the animals were euthanized and the relevant experimental parameters (bodyweight, glycemia, H_2_S levels and others) were assessed comparatively in diabetic dTg mice and as controls, the non‐diabetic sTg and wild‐type mice (sTg and WT). The results showed that the average bodyweight of diabetic group decreased significantly by 1.3‐fold (*P* ≤ .01). More so, the dTg demonstrated a ~3‐fold increase over sTg group (*P* ≤ .001) and 5.6‐fold vs WT (*P* ≤ .001) in plasma glucose concentration (Figure 1A). Moreover, the hyperglycaemic state of the diabetic animals was accompanied by a significant increase in plasma cholesterol compared to the values of the controls. The cholesterol values obtained for dTg were higher by ~1.2‐fold vs WT (*P* ≤ .05) and by ~1.3 vs sTg (*P* ≤ .001) control groups (Figure 1B). Thus, these animals are hyperglycaemic and slightly hypercholesterolaemic, as it is often the case in diabetic patients.

The H_2_S levels were measured in plasma and renal tissue homogenates using the methylene blue method according to the manufacturer's instructions. Interestingly, the results showed that the H_2_S levels in both the renal cortex and plasma increased significantly (by ~3.4‐fold, *P* ≤ .05 and by ~1.9‐fold, *P* ≤ .05, respectively) over the control samples (Figure 1C,D).

### 3.2 Type‐1 diabetes induces subtle structural changes of kidney tissue

**FIGURE 2:**
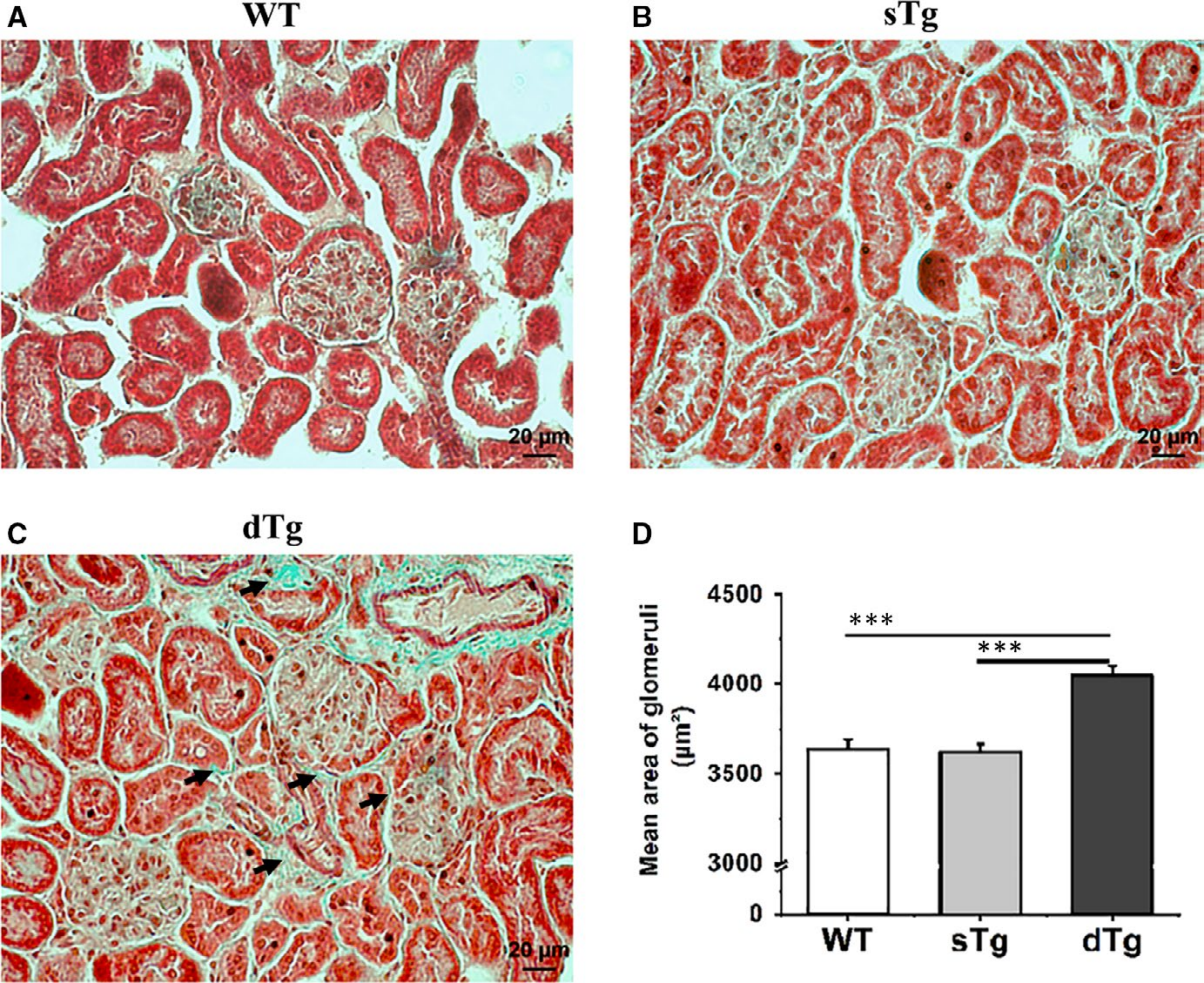
Representative images of Masson's trichrome staining of kidney tissue sections (A‐C). Note that the diabetic kidney tissue exhibits marked interstitial fibrosis (blue stained) surrounding the uriniferous tubules (C, arrows). Morphometric analysis (D) shows that the mean area of glomeruli was significantly increased in diabetic kidney tissue compared to non‐diabetic controls. Data are expressed as mean ± SD; ***P < .001

Staining of renal tissue sections with haematoxylin‐eosin showed no histological abnormalities in the kidney isolated from the control groups (WT and sTg). Diabetic kidney tissue from the dTg group exhibited an apparently normal structure of the glomeruli and the Bowman's capsules, as well. However, the Masson's trichrome staining (Figure 2A‐C) evidenced minor variations in the tubulointerstitial structure of the diabetic kidney. Thus, in the dTg kidney sections we detected the presence of interstitial fibrosis surrounding the uriniferous tubules (Figure 2C, blue staining) and a ~10% (*P* ≤ .001) increase of the mean area of glomeruli compared to control samples (Figure 2D).

### 3.3 Modifications of kidney proteome in T1D revealed by global shotgun proteomic analysis

**FIGURE 3:**
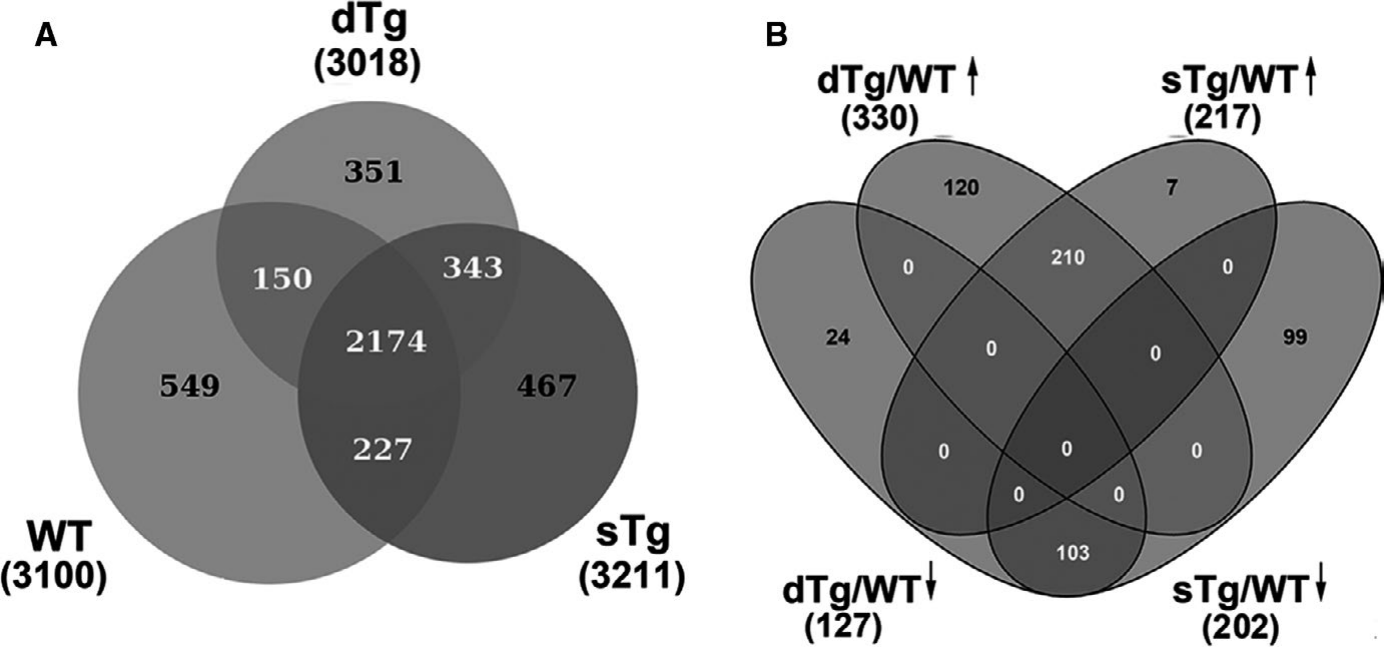
Global proteomic analysis of renal tissue homogenates obtained from double transgenic diabetic mice (dTg), BALB/C wild‐type (WT) and non‐diabetic single transgenic (sTg) mice. (A) Venn diagram revealing the commune and unique proteins identified in diabetic dTg, and controls, WT and sTg. (B) Schematic diagram of differentially abundant proteins up (**↑**) and down (**↓**) regulated in the dTg and sTg groups normalized to WT group. Note that diabetic condition induced changes in 144 proteins abundance as revealed in the diabetic groups dTg/WT (120 up‐ and 24 down‐regulated). Ten sets of experiments were performed, each in technical triplicates

Nano‐liquid chromatography coupled with tandem mass spectrometry (nLC‐MS/MS) experiments were applied on samples of homogenates of renal cortex isolated from dTg, sTg and WT mice. The data led to the identification of 351 proteins that were uniquely identified in dTg, 467 were found only in sTg, while 549 were uniquely attributed to the WT group (Figure 3A). Among all the identified proteins (4261), we also found (average Mascot Score >100) nine of the most frequently studied urinary biomarkers for diabetic nephropathy,^17^ supporting the reliability of our method (listed in Table S1 as supplementary data).

The dTg and sTg proteomes were normalized over the WT mice and the resulting differentially abundant proteins were retained for further comparative analysis (Figure 3B). Thus, 106 proteins (7 up‐regulated and 99 down‐regulated) were found to be uniquely and differentially expressed in sTg/WT, while 144 (120 up‐regulated and 24 down‐regulated) exhibited a significantly altered abundance only in diabetic kidney group, dTg/WT. Finally, 313 proteins (210 up‐regulated and 103 down‐regulated) were common to both groups (Figure 3B). Worth mentioning is that, compared to controls, the majority of proteins (120 out of 144) were up‐regulated in the diabetic group.

Gene ontology analysis of the entire set of 144 differentially abundant proteins induced by diabetic condition revealed that *mitochondrion* (89 proteins)*, small molecule metabolic process* (70 proteins) *and oxidoreductase activity categories* (111 proteins) were statistically over‐represented. The relevant gene ontology classification data (by *Cellular Components, Biological Processes, and Molecular Functions*) for the differentially expressed proteins in diabetic dTg/WT mice are included as supporting information in Table S2 as supplementary data.

**FIGURE 4:**
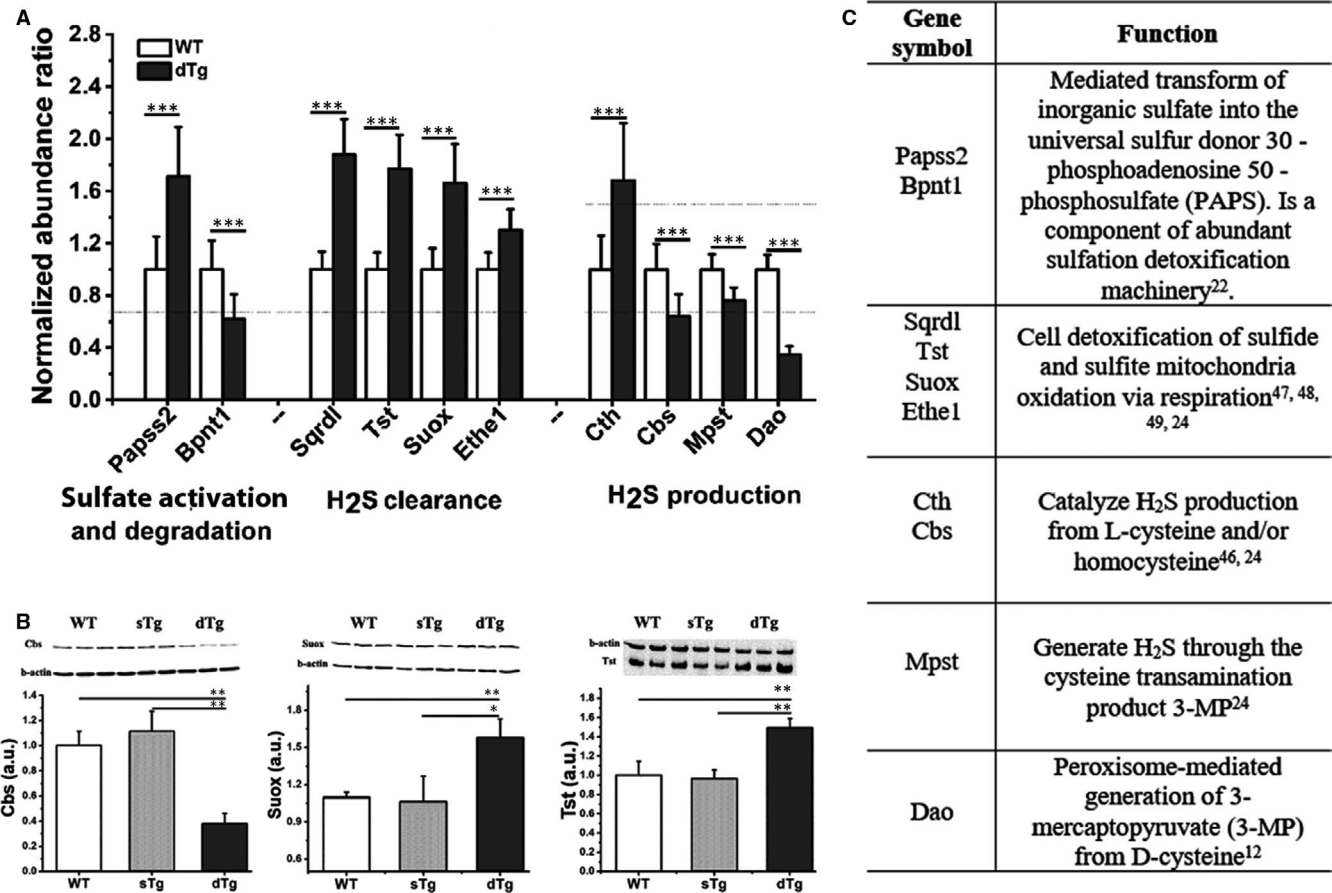
Changes in protein abundance induced by diabetes implicated in KEGG sulphur metabolism pathway and H_2_S production. (A) The proteins involved in sulphate activation and degradation, in clearance and H_2_S endogenous production are separately represented. Note that the following enzymes are up‐regulated in diabetic kidney of double transgenic **(**dTg) samples vs wild‐type (WT) renal homogenates: Papss2 (by ~1.7‐fold, *P* ≤ .001), Sqrdl (by ~1.9‐fold, *P* ≤ .001), Tst (by ~1.8‐fold, *P* ≤ .001), Suox (by ~1.7‐fold, *P* ≤ .001), Ethe1 (by ~1.3‐fold, *P* ≤ .001), Cth (by ~1.7‐fold, *P* ≤ .001). In contrast, decreased abundance was detected for four enzymes (Bpnt1 by ~1.6‐fold, *P* ≤ .001; Cbs by ~1.6‐fold, *P* ≤ .001; Mpst by ~1.3‐fold, *P* ≤ .001 and Dao by ~2.9‐fold, *P* ≤ .001) induced by the transgenic intervention leading towards diabetes. Dashed lines represent the 1.5‐fold regulation factor over the control samples. (B) Validation by Western blot of some protein markers identified by mass spectrometry: cystathionine β‐synthase (Cbs), sulphite oxidase, mitochondrial (Suox) and thiosulphate sulphurtransferase (Tst). Representative immunoblots and densitometric analysis for each enzyme are shown. Statistical analysis was made using Student's *t* test, and the results are expressed as means ± SD; *P < .05; **P < .01; ***P < .01. (C) Table display the references and main functions of enzyme involved in sulphur metabolism identified by the proteomic results. Papps2: bifunctional 3'‐phosphoadenosine 5'‐phosphosulphate synthase 2, Sqrdl: sulphidequinoneoxido reductase, Ethe1: protein Ethe1_mitochondrial; Cth: cystathionine γ‐lyase; Bpnt1: 3(2)5 bisphosphate nucleotidase 1, Mpst: 3‐mercaptopyruvate sulphur transferase; Dao: D‐amino acid oxidase

The quantitative analysis comparing dTg and sTg with WT sets indicated 56 statistically significant differentially abundant proteins (Table S3 as supplementary data) involved in nine statistically over‐represented KEGG pathways (FDR *P*‐value <2.04E‐02) just in the diabetic samples (Table 1). This analysis evidenced ten enzymes related to the sulphur metabolism. Six of them were found to be involved in the first KEGG pathway (mmu00920‐ highest FDR corrected p‐value) and are implicated in *sulphate activation and degradation* and *in H_2_S clearance* and four of them were responsible for the *H_2_S production*. Figure 4A shows the abundance of the enzymes up‐regulated in the diabetic kidney as compared to control: bifunctional 3'‐phosphoadenosine 5'‐phosphosulphate synthase 2 (Papps2), sulphidequinoneoxido reductase (Sqrdl), thiosulphate sulphur transferase (Tst), sulphite oxidase (Suox), protein Ethe1_mitochondrial (Ethe1) and cystathionine γ‐lyase (Cth).

**TABLE 1:** Over‐represented KEGG pathways in diabetic vs non‐diabetic kidney tissue

No.	Description	FDR *P*‐value
1	Sulphur metabolism (mmu00920)	3.46E‐05
2	Proximal tubule bicarbonate reclamation (mmu04964)	5.56E‐05
3	Glycine, serine and threonine metabolism (mmu00260)	6.40E‐05
4	Cysteine and methionine metabolism (mmu00270)	1.08E‐04
5	Arginine and proline metabolism (mmu00330)	3.10E‐03
6	D‐Glutamine and D‐glutamate metabolism (mmu00471)	3.35E‐03
7	Seleno‐compound metabolism (mmu00450)	5.50E‐03
8	Lysine degradation (mmu00310)	1.69E‐02
9	PPAR signalling pathway (mmu03320)	2.04E‐02

In contrast, 3(2)5 bisphosphate nucleotidase 1 (Bpnt1), cystathionine β‐synthase (Cbs), 3‐ mercaptopyruvate sulphur transferase (Mpst) and D‐amino acid oxidase (Dao) were found to be significantly down‐regulated in diabetic kidney (Figure 4A). These enzymes were previously shown to have specific metabolic functions and to be responsible for the bioavailability of sulphate and the H_2_S homeostasis in different experimental models and biological mechanisms (see Figure 4C).

The results reported above for Cbs, Suox and Tst were validated by Western blot and real‐time PCR assays. Thus, the immunoassays revealed the down‐regulation of Cbs (~2.5‐fold, *P* ≤ .01) and the up‐regulation of Suox (~1.6‐fold, *P* ≤ .01) and Tst (~1.5‐fold, *P* ≤ .01) macromolecules in the diabetic kidney (Figure 4B). These results confirmed and were in good agreement with the mass spectrometry data.

**FIGURE 5:**
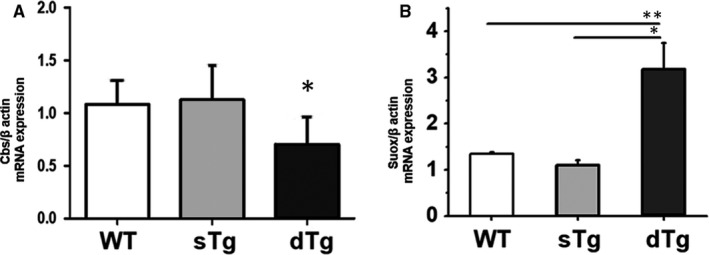
mRNA gene expression level for: (A) cystathionine β‐synthase (Cbs) and (B) sulphite oxidase, mitochondrial (Suox) in kidney homogenate from wild‐type (WT), single transgenic mice (sTg) and double transgenic mice **(**dTg) normalized to b‐actin gene. Note that RT‐PCR analysis revealed the down‐regulation of Cbs gene expression and the up‐regulation of Suox molecule in diabetic renal tissue. The results support the mass spectrometry quantification and immunoassay data. Statistical analysis was performed using Student's *t* test, and the results show means ± SD; *P < .05; **P < .01; n = 5

In addition, the Cbs and Suox gene expression was verified in the kidney homogenates by real‐time PCR. The results back up and support the data obtained by MS and immunoassay, confirming the down‐regulation of Cbs gene expression (by ~1.8‐fold, *P* ≤ .05) and the up‐regulation of Suox molecule (~2.3‐fold, *P* ≤ .01) in diabetic renal tissue (Figure 5A,B).

## 4 DISCUSSION

This study was designed to uncover the debated role of H_2_S in kidney dysfunction that is characteristic for T1D. To this purpose, we took advantage of the availability of the global comparative shotgun proteomic analysis and of the accessibility to a double transgenic mouse model (dTg) which develops T1D. These mice exhibit hyperglycaemia, mild hypercholesterolaemia, weight loss and early kidney dysfunction, the latter revealed by the subtle histological alterations of the kidney tissue. These results corroborate well with previously published data^4^, ^18^, ^19^, ^20^ on dTg mice, which develop autoimmune T1D via a gradual process^3^ associated with low level of plasma insulin and glycosuria. Importantly, this mouse model exhibits characteristics similar to those described in human patients, as ‘near normal diabetic nephropathy’ or Class I diabetic nephropathy.^20^ This is supported by our histological results (Figure 2) demonstrating an early activation of the fibrotic pathways at the kidney level, together with the lower spectral abundance of heparan sulphate proteoglycan 2 and uromodulin (Table S1 as supplementary data). The latter are two important protein markers of early kidney injury,^17^ indicating an uncomplicated diabetes without major damage of renal tissue.

The global gene ontology characterization of differentially abundant proteins revealed their association with mitochondrion and oxidoreductase activity (see Table S2 as supplementary data). These data extend the observations made in diabetic animal models and humans, which state that mitochondrial dysfunction is at the centre of renal disease development and progression.^21^

Using a T1D mouse model and relative quantitative proteomic analysis, we unambiguously revealed the deregulated tissue protein levels of Papss2, Bpnt1, Sqrdl, Tst, Suox and Ethe1 enzymes. These were associated with minor structural changes in the kidney tissue suggesting novel and alternative mechanisms for the generation of early nephropathy in T1D.

LC‐MS/MS results revealed that enzymatic sulphate activation is affected by up‐regulation of Papss2 and the down‐regulation of Bpnt1. The latter removes cytoplasmic Pap, the otherwise toxic by‐product of sulfation. Our data also showed that hyperglycaemia induces a decrease of Bpnt1 protein expression correlated with renal tissue damage, data that concur with studies disclosing that the kidney proximal tubule epithelial cells are affected in the Bpnt1 knockout mouse.^22^

Although often overlooked, a low concentration of hydrogen sulphide has a role in the regulation of physiological homeostasis. Clearance of accumulated toxic levels of hydrogen sulphide in the tissues occurs mainly by mitochondrial oxidation pathways through a respiratory route. This pathway has established hydrogen sulphide as the first non‐carbon‐based respiratory fuel in mammalian systems.^23^, ^24^ In this study, we detected the up‐regulation of mitochondrial Sqrdl, Tst and Suox, Ethe1 enzymes, which are implicated in H_2_S oxidation pathway, one of the hydrogen sulphide clearance mechanisms mentioned above. It is safe to assume that in early diabetic nephropathy, impaired sulphide oxidation, plays an important part in the oxidative stress‐mediated kidney damage. To the best of our knowledge, the results of our proteomic analysis are the first that link impairment of H_2_S oxidation pathway with deregulated mitochondrial enzymes (Sqrdl, Tst and Suox) in early T1D nephropathy.

There are reports indicating a tight correlation between the protein expression of Cth and Cbs with renal tissue H_2_S production induced by diabetes.^3^ Indeed, in our experiments the two enzymes, as well as Mpst and Dao, were found to be significantly down‐regulated in diabetic condition (Figure 4A). Under physiological conditions, the level of renal Cth protein was reported to be ~20‐fold higher than Cbs, suggesting that the former may be the main H_2_S‐forming enzyme in the kidney.^25^ From our data, the differential abundance of selected enzymes (Cth, Cbs, Mpst and Dao), associated with kidney H_2_S production, correlates well with the increased H_2_S level detected in plasma and kidney homogenate (Figures 4 and 1C,D). These data are in good agreement with previous findings stating that H_2_S production is sustained by overexpression of tissue Cth in response to diabetes.^26^ There are reports recognizing that in streptozotocin‐induced diabetic rats, renal H_2_S‐producing enzymes Cbs and Cth are down‐regulated.^10^ ^.^ Our results confirmed the down‐regulation of Cbs, but not Cth protein. This discrepancy could relate to our model, which exhibits early‐stage diabetic kidney disease that differs from advanced chronic nephropathy occurring in streptozotocin‐induced diabetes.

The effect of a high concentration of H_2_S in the plasma and kidney tissue of diabetic patients is controversial. Some reports claim that H_2_S exhibits vascular‐protective effects, causing vasodilatation in diabetes^27^, ^28^ and reducing vascular smooth muscle proliferation.^29^, ^30^ Others describe that H_2_S may promote apoptosis of aortic smooth muscle cells,^31^ and scavenges peroxynitrite^32^ which contribute to the nitrosative stress,^33^ and consequently to endothelial damage associated with this condition.^34^

In our experiments, the T1D mice, non‐obese, non‐overweight and exhibiting an early Class I (like) diabetic nephropathy, exhibited elevated levels of plasma H_2_S (Figure 1C,D), as reported for patients with proliferative diabetic retinopathy ^34^, ^35^ and cardiovascular disease.^36^

It is important to emphasize that the amplified level of H_2_S was present in the renal cortex of T1D animals. This is in contrast to previous data reporting reduced H_2_S content in diabetic acute (formerly known as acute renal failure) or chronic nephropathy.^6^, ^37^, ^38^, ^39^ This discrepancy could be due to the difference between the early stage of diabetic kidney disease without advanced pathological alterations, and the chronic nephropathy characterized by glomerular basement membrane thickening, mesangial matrix deposition and renal interstitial fibrosis.

Several therapies are currently indicated to regulate the abnormal H_2_S content in order to ameliorate advanced renal injury in diabetes.^17^, ^40^ One can envisage the need for therapeutic interventions for the early stages of diabetic nephropathy to delay or halt its progression.

In summary, these experiments showed that the mechanism underlying the elevated renal and plasma H_2_S content in early T1D nephropathy relates to the deregulated—differential expression of specific renal enzymes involved in *sulphate activation and degradation*, *H_2_S production* and *clearance* mechanisms.

Based on these novel findings, we can safely assume that the dysregulation of the enzymes involved in H_2_S metabolism plays a significant part in the aetiology of nephropathy in diabetes and may recommend them as new targets for the therapy of T1D.

## 5 FAIR DATA SHARING

The mass spectrometry proteomics data have been deposited to the ProteomeXchange Consortium via the PRIDE^41^, ^42^, ^43^ partner repository with the data set identifier PXD018053.

## CONFLICT OF INTEREST

The authors declare no conflicts of interest.

## AUTHOR CONTRIBUTION

**Elena Uyy:** Data curation (equal); Formal analysis (equal); Investigation (equal); Methodology (equal); Software (equal); Validation (equal); Writing‐original draft (equal). **Viorel Iulian Suica:** Data curation (equal); Formal analysis (equal); Investigation (equal); Methodology (equal); Software (equal); Writing‐original draft (equal). **Raluca Maria Boteanu:** Data curation (equal); Formal analysis (equal); Methodology (equal). **Florentina Safciuc:** Methodology (equal); Visualization (equal). **Aurel Cerveanu‐Hogas:** Software (equal). **Luminita Ivan:** Methodology (equal). **Crina Stavaru:** Methodology (equal). **Maya Simionescu:** Resources (equal); Writing‐review & editing (equal). **Felicia Antohe:** Conceptualization (equal); Formal analysis (equal); Funding acquisition (equal); Project administration (equal); Resources (equal); Supervision (equal); Writing‐original draft (equal); Writing‐review & editing (equal).

## Supporting information

Tab S1

Tab S2

Tab S3

## Data Availability

Data openly available in a public repository that issues datasets with DOIs.
