# Supplementary material for: Diabetic nephropathy associates with deregulation of enzymes involved in kidney sulphur metabolism
Source: J Cell Mol Med. 2020 Sep 16;24(20):12131–40. doi: 10.1111/jcmm.15855 (PMC7579703; doi:10.1111/jcmm.15855)
Supplement: Supplementary file 1 — Tab S1 [file JCMM-24-12131-s001.docx]

**Supplementary Table S1. General characteristics of nine potential markers for each stage of diabetic nephropathy** identified in kidney tissue isolated from BALB/C wild type (WT), nondiabetic single transgenic (sTg) and diabetic double transgenic mice (dTg). Kidney tissue proteins normalized ratio was expressed as dTg vs. WT and sTg vs. WT. The UniProt database accession codes (UniProt), gene official symbols, median Mascot score and median normalized ratios (including standard deviation and statistical significance) are also presented. ***p < 0.001. UD: Uncomplicated diabetes; IDN: incipient diabetic nephropathy; ODN: Overt diabetic nephropathy.

| **No.** | **Stage of Diabetic Kidney Disease** | **UniProt code** | **Protein description** (Official symbol) | **No. of quantified peptides** | **Mascot score**  **(mean)** | **sTg/WT**  **mean±SD** | **dTg/WT**  **mean ±SD** |
| --- | --- | --- | --- | --- | --- | --- | --- |
| 1 | U.D. | Q05793 | **Heparan sulphate proteoglycan (Hspg2)** | 18 | 38.08 | 0.55±0.06*** | 0.621±0.065*** |
| 2 | U.D | Q91X17 | **Uromodulin (Umod)** | 4 | 331 | 0.71±0.16*** | 0.669±0.14*** |
| 3 | IDN | U3KLX7 | Cubilin (Cubn) | - | 2309 |  |  |
| 4 | IDN | P19134 | Serotransferrin (Tf) | - | 3257 |  |  |
| 5 | ODN | P29699 | α2-HS-glycoprotein (Ahsg) | - | 614 | - | - |
| 6 | ODN | P01887 | β2-microglobulin (B2M) | - | 128.7 | - | - |
| 7 | ODN | Q91X72 | Hemopexin (HPX) | - | 208 | - | - |
| 8 | ODN | Q00724 | Retinol-binding protein 4 (RBP4) | - | 111 |  |  |
| 9 | ODN | P07309 | Transthyretin (TTR) | - | 329.6 |  |  |
