# Supplementary material for: Diabetic nephropathy associates with deregulation of enzymes involved in kidney sulphur metabolism
Source: J Cell Mol Med. 2020 Sep 16;24(20):12131–40. doi: 10.1111/jcmm.15855 (PMC7579703; doi:10.1111/jcmm.15855)
Supplement: Supplementary file 3 — Tab S3 [file JCMM-24-12131-s003.docx]

**Supplementary Table S3**. **General characteristic of the selected proteins** implicated in over-represented signaling pathway in diabetic versus non-diabetic kidney tissue. ***p < 0.001

| **No.** | **UniProt code** | **Protein description** | **No. of peptides quantification** | **Gene**  **official**  **symbol** | **Mascot score**  **(mean)** | **sTg/WT**  **mean±std dev** | **dTg/WT**  **mean ±std dev** |
| --- | --- | --- | --- | --- | --- | --- | --- |
| 1 | Q9R112 | Sulfide:quinone oxidoreductase, mitochondrial | 8 | Sqrdl | 514.14 | 1.63±0.23*** | 1.89±0.27*** |
| 2 | P52196 | thiosulfate sulfurtransferase | 5 | Tst | 642.5 | - | 1.77±0.26*** |
| 3 | O88428 | bifunctional 3'-phosphoadenosine 5'-phosphosulfate synthase 2 | 4 | Papss2 | 571.38 | - | 1.71±0.38*** |
| 4 | Q8R086 | Sulfite oxidase, mitochondrial | 4 | Suox | 773.28 | - | 1.66±0.3*** |
| 5 | Q9Z0S1 | 3'(2'),5'-bisphosphate nucleotidase 1 | 2 | Bpnt1 | 1505.2 | - | 0.62±0.19*** |
| 6 | Q9QZD8 | Mitochondrial dicarboxylate carrier | 7 | Slc25a10 | 640.84 | 1.98±0.36*** | 2.19±0.37*** |
| 7 | O88343 | Electrogenic sodium bicarbonate cotransporter 1 | 4 | Slc4a4 | 894.53 | 1.65±0.28*** | 2.01±0.35*** |
| 8 | P00920 | Carbonic anhydrase 2 | 19 | Ca2 | 2525.16 | 0.37±0.03*** | 0.56±0.04*** |
| 9 | Q9Z2V4 | phosphoenolpyruvate carboxykinase, cytosolic [GTP] | 10 | Pck1 | 2471.06 | - | 2.07±0.29*** |
| 10 | Q8VDN2 | Sodium/potassium-transporting ATPase subunit alpha-1 | 9 | Atp1a1 | 7932.4 | - | 1.99±0.25*** |
| 11 | D3Z7P3 | Glutaminase kidney isoform, mitochondrial | 11 | Gls | 733.79 | - | 1.9±0.22*** |
| 12 | P14152 | Malate dehydrogenase, cytoplasmic | 5 | Mdh1 | 3392.16 | - | 1.66±0.25*** |
| 13 | P26443 | Glutamate dehydrogenase 1, mitochondrial | 30 | Glud1 | 3721.5 | - | 1.47±0.09*** |
| 14 | Q91Z53 | Glyoxylate reductase/hydroxypyruvate reductase | 2 | Grhpr | 1388.83 | 1.95±0.59*** | 1.84±0.53*** |
| 15 | Q9DBT9 | Dimethylglycine dehydrogenase, mitochondrial | 6 | Dmgdh | 1372.5 | 1.58±0.22*** | 1.70±0.23*** |
| 16 | Q8BJ64 | Choline dehydrogenase, mitochondrial | 12 | Chdh | 1643.53 | 1.57±0.16*** | 1.80±0.18*** |
| 17 | Q9DBJ1 | Phosphoglycerate mutase 1 | 7 | Pgam1 | 1770.8 | 0.65±0.07*** | - |
| 18 | Q8QZY2 | Glycerate kinase | 2 | Glyctk | 150.44 | 0.61±0.12*** | - |
| 19 | P18894 | D-amino-acid oxidase | 6 | Dao | 706.02 | 0.4±0.06*** | 0.35±0.06*** |
| 20 | Q8CFA2 | Aminomethyltransferase, mitochondrial | 4 | Amt | 93.69 | - | 1.86±0.35*** |
| 21 | Q3UEG6 | Alanine--glyoxylate aminotransferase 2, mitochondrial | 7 | Agxt2 | 873.16 | - | 1.7±0.19*** |
| 22 | Q8VCN5 | Cystathionine gamma-lyase | 2 | Cth | 1018.51 | - | 1.68±0.44*** |
| 23 | O88986 | 2-amino-3-ketobutyrate coenzyme A ligase, mitochondrial | 6 | Gcat | 477.30 | - | 1.56±0.28*** |
| 24 | Q91WT9 | Cystathionine beta-synthase | 3 | Cbs | 382.56 | - | 0.64±0.17*** |
| 25 | Q9D826 | Peroxisomal sarcosine oxidase | 6 | Pipox | 1686.53 | - | 0.6±0.11*** |
| 26 | P16125 | L-lactate dehydrogenase B chain | 5 | Ldhb | 4256.66 | 1.56±0.29*** | 1.85±0.28*** |
| 27 | O35855 | Branched-chain-amino-acid aminotransferase, mitochondrial | 2 | Bcat2 | 480.90 | 1.46±0.41*** | 1.8±0.49*** |
| 28 | P05202 | Aspartate aminotransferase, mitochondrial | 15 | Got2 | 3324.33 | 0.63±0.07*** | - |
| 29 | P05201 | Aspartate aminotransferase, cytoplasmic | 10 | Got1 | 1486.86 | 0.58±0.07*** | - |
| 30 | P06151 | L-lactate dehydrogenase A chain | 7 | Ldha | 2087.96 | 0.43±0.06*** | 0.46±0.07*** |
| 31 | P08249 | Malate dehydrogenase, mitochondrial | 16 | Mdh2 | 6198.36 | - | 1.51±0.13*** |
| 32 | P47740 | Fatty aldehyde dehydrogenase | 8 | Aldh3a2 | 264.38 | 1.97±0.27*** | 1.91±0.25*** |
| 33 | Q9D1A2 | cytosolic non-specific dipeptidase | 17 | Cndp2 | 2035.7 | 0.51±0.04*** | 0.42±0.04*** |
| 34 | P29758 | Ornithine aminotransferase, mitochondrial | 13 | Oat | 1095.38 | - | 1.81±0.22*** |
| 35 | Q9DCU9 | 4-hydroxy-2-oxoglutarate aldolase, mitochondrial | 5 | Hoga1 | 1215.5 | - | 1.68±0.23*** |
| 36 | Q9WU79 | Proline dehydrogenase 1, mitochondrial | 1 | Prodh | 1541.53 | - | 1.63±0.16*** |
| 37 | Q9CPY7 | cytosol aminopeptidase | 5 | Lap3 | 3538.33 | - | 0.63±0.11*** |
| 38 | Q8CHT0 | Delta-1-pyrroline-5-carboxylate dehydrogenase, mitochondrial | 4 | Aldh4a1 | 2597.36 | - | 0.59±0.13*** |
| 39 | Q9JLI6 | selenocysteine lyase | 2 | Scly | 147.53 | - | 1.69±0.55*** |
| 40 | Q71RI9 | Kynurenine--oxoglutarate transaminase 3 | 5 | Ccbl2 | 708.96 | 0.55±0.12*** | 0.63±0.13*** |
| 41 | P40936 | Indolethylamine N-methyltransferase | 4 | Inmt | 1716.93 | 0.57±0.11*** | 0.59±0.11*** |
| 42 | Q8BTY1 | Kynurenine--oxoglutarate transaminase 1 | 3 | Ccbl1 | 257.2 | 0.67±0.17*** | - |
| 43 | Q60759 | Glutaryl-CoA dehydrogenase, mitochondrial | 8 | Gcdh | 1575.36 | 1.56±0.19*** | 1.86±0.21*** |
| 44 | Q9DBM2 | Peroxisomal bifunctional enzyme | 6 | Ehhadh | 8348.5 | 0.67±0.13*** | 0.45±0.11*** |
| 45 | Q99K67 | Alpha-aminoadipic semialdehyde synthase, mitochondrial | 14 | Aass | 3561.53 | - | 1.91±0.22*** |
| 46 | Q9D2G2 | Dihydrolipoyllysine-residue succinyltransferase component of 2-oxoglutarate dehydrogenase complex, mitochondrial | 18 | Dlst | 1841.2 | - | 1.56±0.12*** |
| 47 | Q60597 | 2-oxoglutarate dehydrogenase, mitochondrial | 26 | Ogdh | 4117.76 | - | 1.54±0.11*** |
| 48 | P51174 | Long-chain specific acyl-CoA dehydrogenase, mitochondrial | 2 | Acadl | 2410.8 | 1.59±0.58*** | 1.97±0.58*** |
| 49 | P97742 | Carnitine O-palmitoyltransferase 1, liver isoform | 3 | Cpt1a | 271.2 | 1.96±0.47*** | 1.86±0.46*** |
| 50 | P52825 | Carnitine O-palmitoyltransferase 2, mitochondrial | 8 | Cpt2 | 1161.63 | - | 1.78±0.24*** |
| 51 | P41216 | Long-chain-fatty-acid--CoA ligase 1 | 8 | Acsl1 | 1009.99 | - | 1.71±0.29*** |
| 52 | Q9QXD1 | Peroxisomal acyl-coenzyme A oxidase 2 | 6 | Acox2 | 1197.33 | - | 0.61±0.11*** |
| 53 | Q9R0H0 | Peroxisomal acyl-coenzyme A oxidase 1 | 3 | Acox1 | 4166.1 | - | 0.6±0.2*** |
| 54 | Q921H8 | 3-ketoacyl-CoA thiolase A, peroxisomal | 2 | Acaa1a | 2115.33 | 0.65±0.16*** | 0.51±0.16*** |
| 55 | O35488 | very long-chain acyl-CoA synthetase | 7 | Slc27a2 | 1916.26 | 0.52±0.11*** | 0.50±0.1*** |
| 56 | Q9EPL9 | Peroxisomal acyl-coenzyme A oxidase 3 | 3 | Acox3 | 2142.06 | 1.45±0.33*** | - |
